# Lifetime age‐related changes in clinical laboratory results, aging clocks and mortality predictors in 2412 Golden Retrievers

**DOI:** 10.1111/acel.14438

**Published:** 2025-01-24

**Authors:** Kevin Perez, Brenna Swafford, Julia Labadie, Alejandro Ocampo

**Affiliations:** ^1^ EPITERNA Epalinges Switzerland; ^2^ Scientific Programs Department Morris Animal Foundation Denver Colorado USA; ^3^ Department of Biomedical Sciences, Faculty of Biology and Medicine University of Lausanne Lausanne Switzerland

**Keywords:** aging, clinical labs, clock, dog, mortality

## Abstract

In this study, we investigated age‐related changes in clinical laboratory data and their association with mortality in dogs from the Golden Retriever Lifetime Study. By analyzing complete blood count (CBC) and biochemistry data from 2′412 Golden Retrievers over 16,678 visits, we observed significant changes during the first 2 years of life and throughout aging. Based on these observations, we developed a biological aging clock using a LASSO model to predict age based on blood markers, achieving an accuracy of *R* = 0.78. Although the biological age clock and pace of aging did not significantly improve mortality prediction, a model incorporating all blood biomarkers showed better predictive power for lifetime (C‐index = 0.763) and 1‐year mortality (AUC = 0.817). Our findings underscore the importance of comprehensive blood analysis for aging and mortality prediction in dogs and open the door for the development of novel methods to investigate aging in companion animals.

## Graphical abstract

We analyzed clinical data from 2412 Golden Retrievers to examine age‐related changes and their association with mortality. A biological aging clock using blood markers achieved *R* = 0.78 but did not improve mortality prediction. A full biomarker model predicted lifetime (C‐index = 0.763) and 1‐year mortality (AUC = 0.817) more effectively. Results highlight the possibility to use comprehensive blood analysis for predicting aging and mortality in dogs.

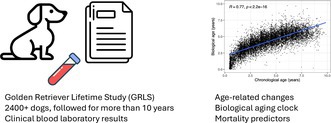

**Abbreviations**
AUC: Area under the curve
CBC: complete blood count
C‐index: Concordance Index
CRP: C‐reactive protein
CV: cross‐validation
GRLS: Golden Retriever Lifetime Study
HCT: hematocrit
HGB: hemoglobin
HR/OR: hazard ratio/odds ratio
LASSO: least absolute shrinkage and selection operator
MCH: mean corpuscular hemoglobin
MCV: mean corpuscular volume
NGS: next‐generation sequencing
r: Pearson correlation coefficient
RBC: red blood cells
RCDW: red cell distribution width
RMSE: root mean square error
WBC: white blood cells

## 1 INTRODUCTION

Although changes in clinical laboratory results (CBC and biochemistry) with age and their association with mortality have been widely studied in humans in large sample size cohorts (Levine et al., 2018), similar studies are lacking for companion animals. In this line, only a few studies have analyzed changes in hematology and blood biochemistry with age with a relatively low sample size (Harper et al., 2003; Lee et al., 2019; Radakovich et al., 2017; Rørtveit et al., 2015). Importantly, dogs have recently become an area of major focus for aging research (Creevy et al., 2022) due to their shorter lifespan and shared socio‐environment with humans in addition to other emotional aspects. For these reasons, it is important to study the biology of aging in dogs, and how it compares to human aging. Moreover, to investigate the effect of therapeutic intervention that could slow down the process of aging and improve healthy lifespan, we need to identify biomarkers of aging (Moqri et al., 2024), which can predict the development of age‐related diseases and all‐cause mortality.

Recently, the development of biological clocks holds such promise (Horvath, 2013). While most current clocks have been developed using next‐generation sequencing (NGS), mainly analyzing DNA methylation, it may be more practical, affordable, and useful to develop clocks built from blood markers routinely used in the clinic (Levine et al., 2018). Indeed, most biological aging clocks developed for dogs to date have relied on DNA methylation (Horvath et al., 2022; Thompson et al., 2017). Importantly, large sample cohorts are necessary to develop aging clocks of high accuracy and predictive power. In this line, the Golden Retriever Lifetime Study (Labadie et al., 2022) (GRLS) from the Morris Animal Foundation is one of the largest, most comprehensive prospective canine health studies in the United States. In this study, we used the GRLS CBC and biochemistry data from more than 2000 Golden Retrievers and 15,000 visits to analyze the changes in blood parameters with age, develop a biological aging clock, and assess its capacity to predict all‐cause mortality.

## 2 RESULTS

### 2.1 Study description

As part of The Golden Retriever Lifetime Study (Labadie et al., 2022) from the Morris Animal Foundation, 2412 neutered Golden Retrievers (1117 male—46%, 1295 female—54%) were followed from June 1st, 2012, to April 1st, 2023. The dogs were seen at participating clinics once a year, with a median number of visits of *N* = 8 (SD 1.9), leading to a median follow‐up of 7 (SD 1.8) years. The age distribution ranged from 0.4 years old to 11 years old, with a median age of 4.4 years old (SD 2.4). The median age at first visit was 1.1years old (SD 0.6). Blood clinical biochemistry and CBC were available for a total of 16,678 visits.

### 2.2 Analysis of changes during development and during aging

**FIGURE 1:**
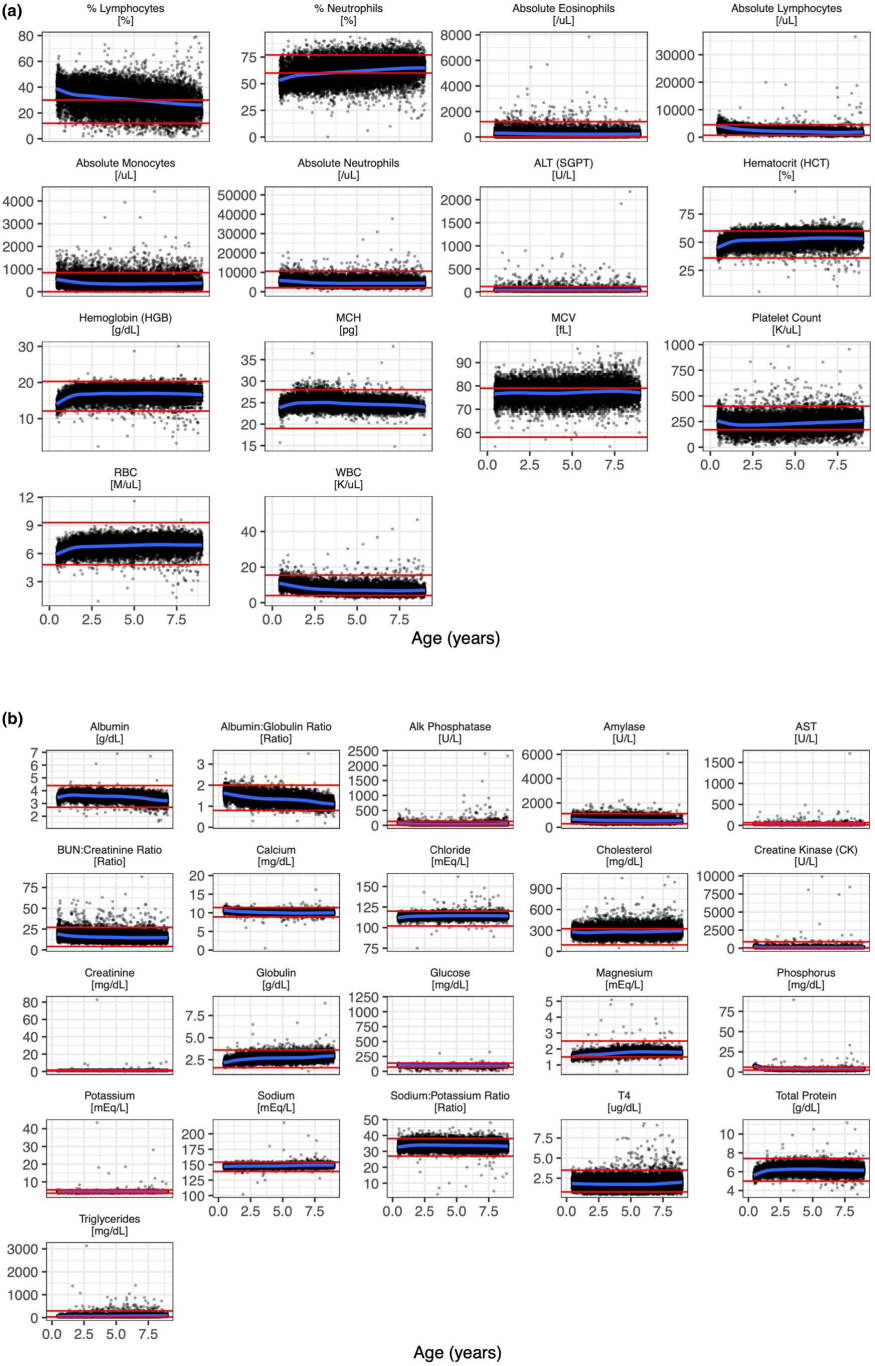
Changes in blood markers with age during development and aging. Changes in complete blood count (CBC) and biochemistry markers during development and aging (a) CBC (b) Biochemistry. Reference ranges in red. In blue, smoothed fit (LOESS).

After accessing the GRLS data, we first analyzed changes occurring during the first years of life of the dogs. Towards that goal, we empirically defined the development phase as the first 2 years of the dog's life, since we observed that most values started to stabilize after 2 years. Notably, the most significant changes we observed during the first 2 years of life were a decrease in phosphorus (*r* = −0.81), alkaline phosphatase (*r* = −0.57), absolute lymphocytes (*r* = −0.45) and calcium (*r* = −0.39); and an increase in hemoglobin (*r* = 0.53), total protein (*r* = 0.48), hematocrit (*r* = 0.41), RBC (*r* = 0.41), globulin (*r* = 0.41) and magnesium (*r* = 0.38, Pearson, *p* < 0.001 for all, Figure 1, Data S1). We next studied changes that occurred during aging, after this initial development phase of 2 years, until death. Interestingly, aging was marked by a significant decrease in albumin (*r* = −0.42), albumin: globulin ratio (*r* = −0.4), absolute lymphocytes (*r* = −0.26), “% lymphocytes” (*r* = −0.24), calcium (*r* = −0.2) and MCH (*r* = −0.2); and an increase in magnesium (*r* = 0.31), globulin (*r* = 0.25), sodium (*r* = 0.24) and “% neutrophils” (*r* = 0.2, Pearson, *p* < 0.001 for all, Figure 1, Data S1). We also analyzed aging associations in each gender separately and observed a high correlation between the changes seen in males and in females (Data S1).

### 2.3 Development of a biological aging clock

**FIGURE 2:**
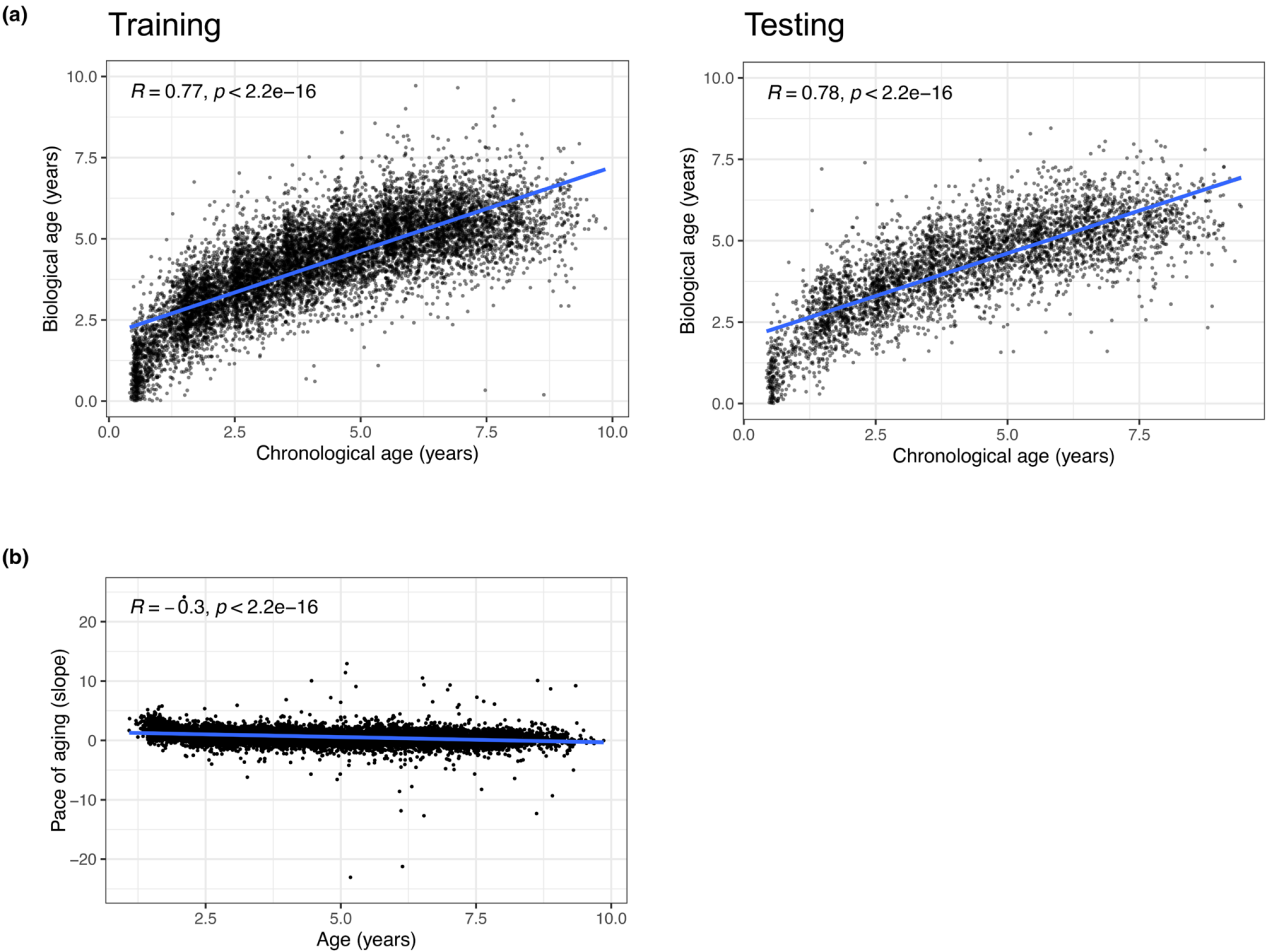
Biological aging clock, pace of aging, and mortality. (a) Performance of biological aging clock from all blood markers in the training and testing set. (b) Pace of aging (slope) as a function of age.

After observing these changes in blood biomarkers during aging, we hypothesized that a biological aging clock could be built for dogs based on these blood changes. For this reason, after splitting the data into 70% training (11,787 visits, 1688 dogs) and 30% testing (4927 visits, 724 dogs) sets, we fit a least absolute shrinkage and selection operator (LASSO) model on the training data to predict chronological age and tested its accuracy on the testing set. Importantly, we reached an accuracy of *R* = 0.78, RMSE = 1.37 years in the training set, and *R* = 0.78, RMSE = 1.38 years in the testing set for predicting age based on blood markers (Figure 2). The most important variables in the model were magnesium, sodium, albumin, calcium and albumin: globulin ratio.

### 2.4 Mortality prediction based on blood biomarkers

Using this newly developed biological aging clock, we sought to test its ability to predict all‐cause mortality in the GRLS study. In this regard, 775 (33%) dogs died during the follow‐up of the study. To evaluate the capacity of the clock to predict mortality, we split the cohort into 70% training (8580 visits, 1632 dogs, 528 deaths—32%) and 30% testing (3589 visits, 700 dogs, 247 deaths—35%) set. Subsequently, we tested the ability of blood markers to predict both lifetime mortality and 1‐year mortality. For lifetime mortality, we fitted a Cox proportional hazard model using 4 different sets of variables: Age alone (i), Age + Biological age (ii), Age + Pace of aging (iii), Age + All blood markers (iv). For 1‐year mortality prediction, we fitted a generalized linear model (GLM) with the same 4 sets of variables. The pace of aging (Belsky et al., 2015) was defined as the slope of biological aging between two consecutive visits. The pace of aging was accelerated during development compared to the adult phase, which is to be expected since dogs are in a period of rapid growth. The results are summarized in Table 1. Importantly, age alone reached a predictive power of Cindex = 0.758 for lifetime mortality and AUC = 0.768 for 1‐year mortality prediction in the testing set. Although biological age and pace of aging alone were not able to improve this mortality prediction, a model including all the blood markers was able to improve the predictive power, particularly for 1‐year mortality prediction, reaching C‐index = 0.763 for lifetime mortality and AUC = 0.817 for 1‐year mortality in the testing set. Specifically, higher amylase was associated with a higher risk of mortality in both the lifetime (HR = 1.12) and 1‐year (OR = 1.34) mortality predictors (Data S1). Of note, female dogs lived longer on average than male dogs (*p* = 0.002), and gender was an important predictor in all the lifetime mortality models, but not in the 1‐year mortality models. These results suggest that a mortality predictor based on all blood markers was better able to predict mortality compared biological age.

**TABLE 1:** Performance of mortality predictors. Performance of the 4 models tested on 1‐year mortality (AUC) and lifetime mortality prediction (C‐index), on both the training and testing sets.

	1‐year mortality (AUC)		Lifetime mortality (C‐index)	
	Train	Test	Train	Test
Age (i)	0.776	0.768	0.769	0.758
Age + Biological age (ii)	0.776	0.767	0.769	0.757
Age + Pace of aging (iii)	0.774	0.773	0.769	0.758
Age + All blood markers (iv)	0.831	0.817	0.777	0.763

### 2.5 Cause‐specific mortality

We next wanted to assess how particular diseases were affecting changes in blood markers in dogs, and their association with cause‐specific mortality. The most tracked conditions in the dogs that died during the study were hemangiosarcoma (24%), mast cell tumors (17%) and lymphoma (13%). Surprisingly very little deaths were attributed to trauma or accident (less than 1%). We first assessed if there were any blood changes in the dogs with these three conditions. While mast cell tumors and lymphomas were not associated with significant blood changes, the presence of hemangiosarcoma was strongly associated with a low platelet count (thrombocytopenia) in the dogs (*p* < 0.001), a finding which has been reported before (Hammer et al., 1991). We next looked at the association between blook markers and cause‐specific mortality. Interestingly, while high amylase was still the best predictor for mortality with Hemangiosarcoma (p < 0.001), it was not a good predictor of mortality with Lymphoma or with Mast cell tumor. This suggest that there may be alternative pathways involved with the mortality associated with these three conditions in the dogs.

## 3 DISCUSSION

During the first 2 years of dogs' life, we find significant changes in blood parameters, notably a decrease in phosphorus, alkaline phosphatase, absolute lymphocytes and calcium; and an increase in hemoglobin, total protein, hematocrit, RBC, globulin and magnesium. Similarly, other groups have previously reported changes in blood during the first years of life. Rørtveit et al. (2015) analyzed the blood of 101 mixed‐breed puppies, up to 60 days of age, and observed a decrease in phosphorus and calcium; and an increase in RBC, HGB, HCT, and globulin. Importantly, we have identified during our study that these values do not stabilize at 2 months of age and continue to change up to 2 years of age. In addition, Harper et al (2003) analyzed the blood of 34 Beagles and 44 Labrador Retrievers. Once again, they also observed, during the first year of life, an increase in RBC, HGB, HCT, total protein and globulin, and a decrease in phosphorus, Alk phosphatase and calcium. Interestingly, according to the 2019 AAHA Canine Life Stages guidelines, a dog's puppy stage last 6–9 months, and the dog is considered as a young adult until 3 to 4 years of age (Creevy et al., 2019). Overall, we observed significant cellular and biochemical changes in blood during the first 2 years of life, consistent with the existing literature (Table 2).

**TABLE 2:** Changes in blood markers with age during development and aging. Increase or decrease in blood CBC and biochemistry markers during development and aging in our study compared to the existing literature.

	Development (0—2 years)			Aging (2—11 years)				
	Perez et al. (2024)	Rørtveit et al. (2015)	Harper et al. (2003)	Perez et al. (2024)	Lee et al. (2019)	Radakovich et al. (2017)	Harper et al. (2003)	Strasser et al. (1993)
Albumin				Decr.		Decr.		Decr.
Alk phosphatase	Decr.		Decr.					
Calcium	Decr.	Decr.	Decr.	Decr.				
Globulin	Incr.	Incr.	Incr.	Incr.		Incr.		
Glucose					Decr.			Incr.
HCT	Incr.	Incr.	Incr.			Decr.		
HGB	Incr.	Incr.	Incr.					
Iron						Decr.		
Lymphocytes	Decr.			Decr.				Decr.
Magnesium	Incr.			Incr.				
MCH				Decr.				
MCV						Decr.		
Neutrophils				Incr.				
Phosphorus	Decr.	Decr.	Decr.					
Platelet						Incr.		Incr.
RBC	Incr.	Incr.	Incr.					
Sodium				Incr.				
Total protein	Incr.		Incr.		Incr.	Incr.		
Urea						Incr.		

After the initial 2‐year period of development, values stabilized, and age‐associated hematological changes were observed during adulthood and aging. Notably, a significant decrease in albumin, albumin: globulin ratio, absolute lymphocytes, “% lymphocytes”, calcium and MCH; and an increase in magnesium, globulin, sodium and “% neutrophils”. Although several groups have investigated changes in hematological and biochemical blood parameters in dogs, these studies have used a much lower sample size. In this line, Lee et al. (2019) found an increase in total protein and a decrease in glucose in 14 Beagles and 14 Retrievers. Similarly, Radakovic et al. (2017) found that HCT, MCV, iron and albumin decreased with age, while total proteins, globulins, platelet and urea increased with age in multiple breeds. Strasser et al. (1993) found a decrease in lymphocytes and albumin, and an increase in glucose, platelets and cortisol with age in 40 German Shepherds and 15 Beagles. On the other hand, Harper et al. (2003) did not identify any significant changes with age after the first year of life, in 34 Beagles and 44 Labrador Retrievers. Overall, we observed significant changes during aging, some of which were previously reported in the literature while others are novel, and importantly on a much larger sample size (Table 2). Of note, other studies have investigated in more detail changes in subtypes of immune cells in dogs with aging, but this type of data was not accessible in our study for comparison. Indeed, Greeley et al. (2001) conducted an 8‐year longitudinal study on 23 Labrador Retrievers (Greeley et al., 2001), and Reis et al. (2005) evaluated age‐related phenotypic changes in canine whole blood leukocytes using 40 mongrel dogs (Reis et al., 2005).

In this study, we were able to develop a novel biological aging clock from routine clinical laboratory tests with high accuracy (*R* = 0.78, RMSE = 1.37y). Other groups have previously built biological clocks for dogs, mainly using DNA methylation. Thompson et al. (2017) built a clock from DNA‐methylation in dogs (*N* = 42) and wolves (*N* = 62). Horvath et al. (2022) built a highly accurate clock (*R* = 0.97) for both dogs and humans with DNA‐methylation using 742 samples from mixed dog breeds. Jin et al. (2024) built a biological age clock using both DNA methylation and chromatin accessibility, but with relatively low accuracy (*R* ^2^ = 0.26, 0.29 or 0.33). Wang et al. (2020) also built a clock from 104 Labrador methylomes, conserved between dogs and humans. As expected, our clock may be less accurate than the most precise DNA methylation clocks, but more clinically usable and affordable. Furthermore, we showed that a mortality predictor based on blood markers was better able to predict mortality than biological age and pace of aging. To date, the predictive power for mortality of aging clocks in dogs had not been assessed thoroughly. Notably, Horvath et al. could not test for mortality prediction due to a lack of follow‐up data available (Horvath et al., 2022). Our results are in line with other results in humans, showing that a clock designed to predict mortality, instead of age, may be a better surrogate marker for biological aging (Lu et al., 2022).

Interestingly, cholesterol and blood glucose did not change with age in dogs, contrary to in humans (Yi et al., 2017, 2019). In humans, Levine et al. (2018) also built a mortality predictor using blood CBC and biochemistry, with a similar method to our model (iv): Age + All blood markers. Interestingly, they found that this clock was associated with increased mortality (HR = 1.09), but did not report the concordance index for mortality prediction. The clock was built on an increase in creatinine, glucose, CRP, MCV, RCDW, alkaline phosphatase and WBC, and a decrease in albumin and lymphocytes. Overall, the changes in blood with age in dogs do not seem to mirror that of humans, and may indicate, to some extent, a distinct physiology or biology of aging in dogs, compared to humans.

The strength of this study lies in the large sample size, homogenous population, data processing, analysis and collection. We also benefit from a wide age range, allowing continuous age analysis as opposed to discrete. In addition, we benefit from a long follow‐up with repeated sampling and extensive mortality data. On the other hand, this study could be improved by future validation in external cohorts. Moreover, this study is limited to one breed, Golden Retriever, and it would be interesting to assess how these results translate to other dog breeds. Overall, our data demonstrates that aging clocks and mortality predictors might be useful during veterinary clinical practice as well as for the investigation of the biology of aging and the development of therapeutic interventions aiming at improving health at old age in companion dogs.

## 4 METHODS

### 4.1 Data acquisition and processing

We acquired data from Data Commons of the GRLS study, provided by the Morris Animal Foundation. Non‐neutered dogs were excluded from the study. We collected all the blood CBC and clinical chemistry data available. CBC was run on a Siemens Advia, and chemistry was run on Beckman Coulter AU‐series for the whole duration of the study. CBC and chemistry were run in one lab from 2012 to Dec. 2020, and another lab from 2021 to April 2023. During this change, a bridging study where samples from 50 dogs were run at both labs (Labadie et al., 2022), confirmed that the change did not impact the results. We kept only variables with less than 10% of missing values across the whole cohort. The magnesium variable was reported using 2 different units, depending on the date the sample was processed (before of after Dec. 2020). For consistency, we kept only the value in “mEq/L.”

### 4.2 Aging correlations

Development and aging changes were computed using the Pearson correlation coefficient (*r*), and the *p‐*values were calculated from a *t*‐statistic.

### 4.3 Biological age clock

To build a biological age clock from the blood parameters we randomly split the data into 70% training (11,787 visits, 1688 dogs) and 30% testing (4927 visits, 724 dogs) sets, fit a least absolute shrinkage and selection operator (LASSO) model on the training data to predict chronological age and tested its accuracy on the testing set. Importantly, each dog and their multiple visits were part of either the training or the testing set, but not spread between both, to avoid training data contamination. The random sampling was therefore done on the dogs, and not on the (dog, visit) data pairs. We used 10‐fold cross‐validation (CV) in the glmnet R package for the training. The accuracy was assessed with R Pearson coefficient and root mean squared error (RMSE).

### 4.4 Pace of aging

The pace of aging was calculated for each visit, starting from visit 2, as the slope of biological aging that is [(Biological Age_v(*n* + 1)−Biological Age_v(*n*))/(Age_v(*n* + 1)−Age_v(*n*))].

### 4.5 Mortality prediction

For mortality prediction, we selected dogs that had at least 2 visits and randomly split the cohort into 70% training (8580 visits, 1632 dogs, 528 deaths—32%) and 30% testing (3589 visits, 700 dogs, 247 deaths—35%) set. Each dog, and their multiple visits, were part of either the training or the testing set, but not spread between both. We tested the ability of blood markers to predict both lifetime and 1‐year mortality. For lifetime mortality, we fitted a Cox proportional hazard model using 4 di/erent sets of variables: Age alone (i), Age + Biological age (ii), Age + Pace of aging (iii), Age + All blood markers (iv). Dogs lost to follow‐up or withdrawn were right‐censored. For 1 year mortality prediction, we fitted a generalized linear model (GLM) with the same 4 sets of variables. All our models were adjusted for gender.

### 4.6 Cause of death

For the association between specific conditions and blood markers, the analysis was conducted on the last timepoint available for the dogs, and adjusted for age, sex and mortality status. For the association between blook markers and cause‐specific mortality, a separate Cox model was used for each specific disease indication.

## AUTHOR CONTRIBUTIONS

K. P. designed the study, performed data analysis and visualization, and co‐wrote the manuscript. B. S., J. L. critically revised the manuscript and aided with data access and analysis. A. O. designed the study and co‐wrote the manuscript.

## FUNDING INFORMATION

This study was funded by EPITERNA.

## CONFLICT OF INTEREST STATEMENT

The authors do not declare any competing interest in the current study.

## Supporting information


Data S1.

## Data Availability

All data produced in the present work are contained in the manuscript. No additional data available. Morris Animal Foundation granted EPITERNA (Kevin Perez) access to data from the GRLS study.
